# Study on Calibration Tests for Interface-Type Earth Pressure Cell Based on Matching Error Analysis

**DOI:** 10.3390/s24237778

**Published:** 2024-12-05

**Authors:** Mingyu Li, Longwei Zhu, Jicheng Shu, Zhenzhen Lu, Yunlong Liu

**Affiliations:** 1 School of Civil Engineering, Zhengzhou University, Zhengzhou 450001, China; mingyu_li@zzu.edu.cn (M.L.); 16627550853@126.com (L.Z.); vakwqt89693@outlook.com (Z.L.); 2 China Railway 14th Bureau Group Corporation Limited, Nanjing 211899, China; 414730970@126.com

**Keywords:** geotechnical engineering, earth pressure, single-membrane resistive earth pressure cell, embedded, interfaced, calibration test, matching error

## Abstract

The stress status of a soil pressure cell placed in soil is very different from its stress state in a uniform fluid medium. The use of the calibration coefficient provided by the soil pressure cell manufacturer will produce a large error. In order to improve the measurement accuracy of the interface-type earth pressure cell placed in soil, this paper focuses on a single-membrane resistive earth pressure cell installed on the surface of a structure, analyzing the influence of loading and unloading cycles, the thickness and particle size of the sand filling, and the depth of the earth pressure cell inserted in the structure on the calibration curve and matching error, which were analyzed through calibration tests. The results show that when the sand filling thickness is less than D (D is the diameter of the earth pressure cell), the calibration curve is unstable in relation to the increase in the number of loading and unloading cycles, which will cause the sand calibration coefficient used for stress conversion to not be used normally. When the sand filling thickness in the calibration bucket increases from 0.285D to 5D, the absolute value of the matching error first decreases and then increases, such that the optimal sand filling thickness is 3D. The output of the earth pressure cell increases with the decrease in sand particle size under the same load, and there is a significant difference between the theoretical calculation value and the experimental value of the matching error; aiming at this difference, an empirical formula is derived to reflect the ratio of the diameter of the induction diaphragm of the earth pressure cell to the maximum particle size of the sand filling. When the depth of the earth pressure cell inserted in the structure is “0”, the sensing surface is flush with the structure and the absolute value of the matching error is the smallest. Changes in the horizontal placement of the soil pressure cell in the calibration bucket result in significant differences in both the output and hysteresis of the calibration curve. To improve the measurement accuracy of soil pressure cells in scaled tests for applications such as in the retaining walls of excavation pits, tunnel outer surfaces, pile tops, pile ends, and soil pressure measurements in soil, calibration of the soil pressure cells is required before testing. Due to the considerable difference in the stress states of the soil pressure cell between granular media and uniform fluid media, calibration in soil is essential. During in-soil calibration, factors such as cyclic loading and unloading, soil compression, sand thickness and particle size, and the placement of the soil pressure cell all affect the calibration results. This paper primarily investigates the influence of these factors on the calibration curve and matching error. This study found that, as the sand thickness increases, the matching error decreases initially and then increases.

## 1. Introduction

Soil pressure measurement is an important method in soil pressure testing research and computational theory, typically involving the use of soil pressure cells to measure soil pressure. The single-membrane resistive micro soil pressure cell is a non-translational type of soil pressure sensor that responds to the deflection of its sensing surface. Due to its small size, high sensitivity, stable readings, and durability, it is widely used in various scaled model tests [1].

Given the diverse test conditions and soil properties in different scaled model experiments, there is often a significant discrepancy between the measured and actual soil pressure values. Therefore, prior to scaled model testing, soil pressure cells generally need to be calibrated [2]. A common method for calibration is to place the soil pressure cell in a fluid medium (oil or gas) [3]. However, since the mechanical properties of fluid media differ significantly from those of granular media (such as soil), researchers have increasingly attempted to calibrate soil pressure cells directly within the soil to avoid measurement errors during the experiment. In studies on soil pressure cell calibration within soil, researchers have found that the loading curve can exhibit hysteresis [4] and that factors such as soil arching effects, sand filling thickness, the grain size of the sand and gravel, installation angle, and soil moisture content can all influence calibration results.

Currently, research on the optimal sand filling thickness for calibration is ongoing. Trllope [5] from Australia was the first to propose the concept of an optimal sand filling thickness for soil pressure cells. Cityhara and Furukawa [5] in Japan derived that the optimal filling thickness should be 0.9 times the effective diameter of the soil pressure cell or 60 times the height-to-diameter ratio (H/D). Subsequently, Zhu et al. [6] and Dave et al. [7] found the optimal sand thickness to be 13.85D and 1.5D, respectively. Additionally, regarding the effect of sand filling thickness on output, Li Chunlin et al. [8], through modified direct shear tests, found that increasing sand thickness reduces the output of the soil pressure cell. On the other hand, Gade et al. [9,10] and Cai Zhengyin et al. [11] observed that increasing the sand thickness increased the output, while dave et al. [7] found that the output increased initially and then decreased with increasing sand thickness. Therefore, the effect of sand thickness on the output of the soil pressure cell remains controversial, warranting further investigation.

In addition to studying sand filling thickness, a few researchers have studied other factors such as gravel particle size, installation angle, and soil moisture content. Kinya et al. [12] found that increasing gravel grain size reduces the contact points for stress between the gravel particles and the sensing diaphragm of the soil pressure cell, thereby decreasing the output. Li Yankun et al. [13] observed that the calibration factor for a laterally embedded soil pressure cell in sand differs significantly from that in oil. Pytka [14] concluded from experiments that increasing soil moisture content makes the output of the soil pressure cell more sensitive to changes, and that the calibration factor is more significantly affected by moisture content in natural soils than in sand and loess. Wang et al. [15,16] found that soil pressure cells buried in over-consolidated and under-consolidated soils exhibit different measurement errors. The analysis of these factors has contributed to the development of research on soil pressure cell calibration in soil. However, due to the limited number of studies, further experimental verification is needed to validate existing research findings, and these results need to be incorporated into computational theories.

Zeng Hui et al. [17] proposed a formula for calculating matching error based on experimental data, which examines the correlation between the structural modulus of elasticity, soil strain, and soil pressure cell diameter and matching error. However, the studies lacked a consideration of the relationship between sand filling thickness and maximum grain size and matching error. Through previous and current experimental research, these factors have been found to have significant correlations with matching error.

In summary, to further validate the correctness of existing research on sand filling thickness, grain size, and spacing between soil pressure cells, and to expand the analysis of the testing effects of soil pressure cells embedded at different depths within test components, this study aims to refine the current computational theory for matching errors. The ultimate goal is to enhance the accuracy of soil pressure measurements in scale model tests investigating the interaction between the excavation of foundation pits and existing underground structures.

## 2. Experimental Protocol Design

### 2.1. Experimental Program

The earth pressure box is embedded in the plexiglass plate and then evenly filled with a covering of sand medium, placed on the loading plate, and a load is applied. Four trials were conducted:

(1) Different upper sand filling thickness test. The earth pressure box is embedded in the groove of the plexiglass plate so that the sensing surface is level with the upper surface of the glass plate structure. The influence of the sand filling thickness on the calibration curve was studied by changing the thickness of the overlying sand layer. The sand filling thickness of the upper and lower parts of the earth pressure box was changed at the same time as the embedded type, and the embedded type changed the sand filling thickness of the upper and lower parts of the earth pressure box at the same time.

(2) Different sand filling particle size test. Four groups of screened sand with different particle sizes were used as the calibration medium, but the sand filling thickness (3D) remained unchanged.

(3) Different horizontal burial position test. The position of the earth pressure box on the plexiglass plate was changed, and the influence of the horizontal distribution position of the interface earth pressure box on its calibration results was analyzed.

(4) Different embedding depth experiments. The depth of the earth pressure box embedded in the groove of the plexiglass plate was changed so that the relative height of the sensing surface of the earth pressure box and the upper surface of the plexiglass plate changed while the sand filling thickness (3D) remained unchanged.

### 2.2. Materials Used in the Study

**Figure 1:**
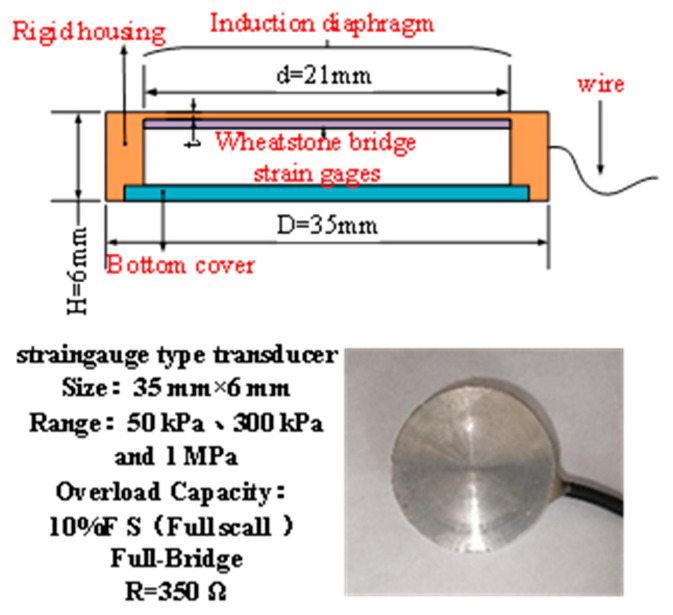
The earth pressure cell.

This experiment used the DYHC-type single-membrane resistive micro soil pressure cell with a measuring range of 300 kPa. The photograph and structural details are shown in Figure 1. The soil pressure cell had a diameter (D) of 35 mm, a thickness (H) of 6 mm, and a thickness-to-diameter ratio (H/D) of 0.17. The diaphragm had a diameter (d) of 21 mm and a thickness (t) of 0.55 mm.

**Figure 2:**
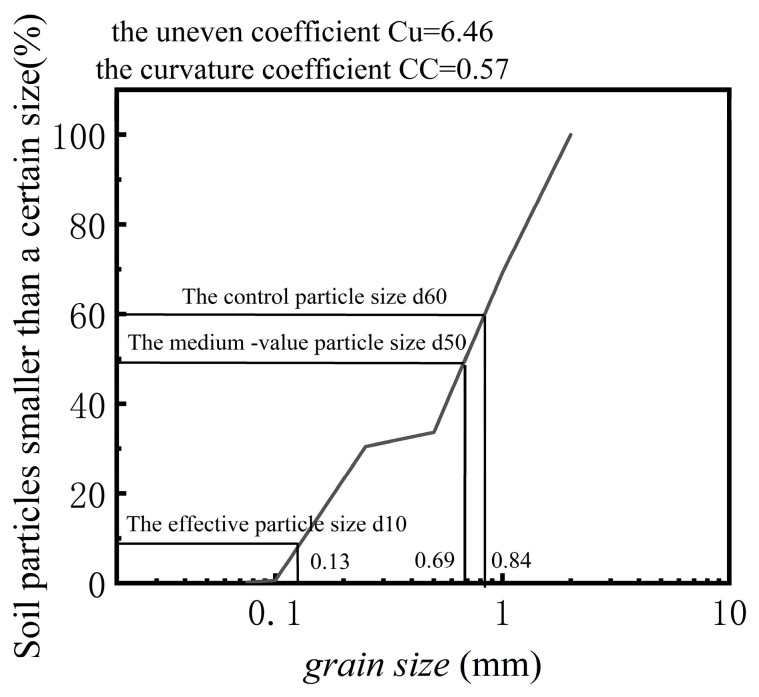
Particle gradation curve of standard sand.

Standard sand was used as the calibration medium. It was produced by China Xiamen Aisou Standard Sand Co., Ltd. (Xiamen, China), as shown in Figure 2.

**Figure 3:**
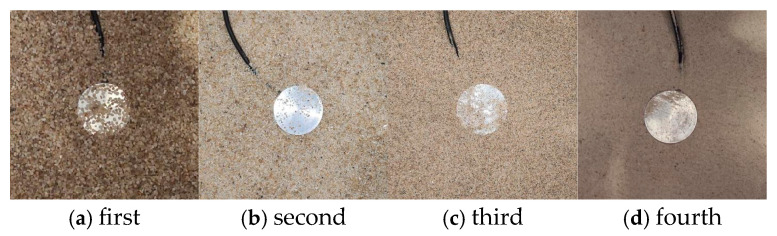
Earth pressure cells in 4 groups of sand with different particle sizes.

In Experiment (2) of Table 1, the standard sand was sieved into four particle size ranges: 1 mm–2 mm, 0.5 mm–1 mm, 0.25 mm–0.5 mm, and finer than 0.25 mm. Arranged from largest to smallest particle size, the corresponding compression moduli are 71 kPa, 28 kPa, 105 kPa, and 107 kPa. Photographs of the soil pressure gauges embedded in the sands of four different particle sizes are shown in Figure 3.

### 2.3. Experimental Setup

**Figure 4:**
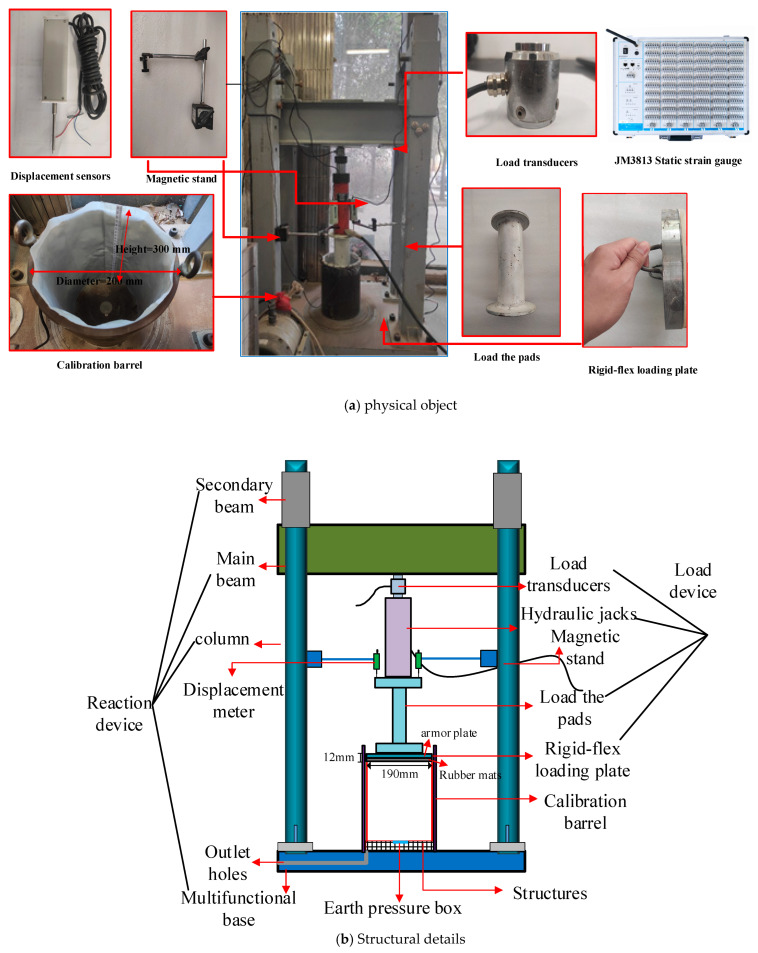
Soil calibration equipment for miniature earth pressure cells.

The entire experimental setup included the reaction frame, calibration barrel, loading system, and soil settlement measurement system, as shown in Figure 4a,b. The reaction frame consisted of the main beam, secondary beam, columns, and base. The main and secondary beams can be adjusted for height, while the base is equipped with slots of varying diameters to accommodate calibration barrels of different sizes. By appropriately increasing the inner diameter of the calibration barrel, the soil arching effect can be effectively reduced. Considering factors such as fill thickness, barrel wall friction, soil arching effect, and the number of soil pressure cells being calibrated, the steel calibration barrel was designed with a height of 300 mm, an inner diameter of 200 mm, and a wall thickness of 12 mm. The loading system comprises a force sensor, a hydraulic jack, a loading pad, and a loading plate. Following the design concept of Jiang Mingjie et al. [18], the loading plate was composed of a steel plate and a rubber pad, with a thickness of 12 mm and a diameter of 190 mm. Additionally, two displacement sensors were mounted on the loading pad during the experiment to measure the vertical compression deformation of the sand and to assist in evaluating the accuracy of the soil pressure measurements. A JM3813 multifunctional static strain acquisition instrument was used to collect data from the soil pressure cells, force sensors, and displacement sensors during the experiment.

### 2.4. Experimental Procedure

1. Embedded Test Procedure

(1) Installation of Calibration Barrel: The calibration barrel was placed onto the base.

(2) Reducing Friction on Barrel Walls: During loading, the side resistance between the inner wall of the calibration barrel and the sand affects the stress distribution of the sand and generates the soil arching effect, which interferes with the test results. To address this, the method of friction reduction from Rui Rui et al. [19] was adopted. First, a layer of polyethylene film was adhered to the inner wall of the calibration barrel using double-sided tape. Then, a layer of graphite was evenly applied on top of the film using a brush. Finally, a second layer of polyethylene film was added. This outer layer of film was not fixed with tape, allowing it to slide freely between the two layers. The film and graphite combination ensures sufficient lubrication, and the second layer of film can be replaced at any time, avoiding wear on the lubricant caused by the sand while enabling reuse.

(3) Filling the Sand: Sand was filled before placing the soil pressure cell, and then sand was added again. The sand was added in layers of 5 mm at a time, with the surface leveled using a spirit level after each 5 mm of sand was added to ensure uniform filling.

(4) Placement of the Soil Pressure Cell: According to the experimental study by Li Yankun et al. [20], the lateral, reverse, and inclined placement of the soil pressure cell can affect the calibration results. Therefore, before placing the soil pressure cell, the center of the calibration barrel was first located using a steel ruler. The soil pressure cell was then placed with the sensing surface facing upward and levelled using a spirit level to ensure that the sensing surface is horizontal. The lead wires were fixed to the side wall of the calibration barrel and led out of the barrel.

(5) Installation of the Loading System: The bottom surface of the loading plate was cleaned and placed on the surface of the filled sand, which was levelled using a spirit level. Then, the loading pad, hydraulic jack, and load sensor were sequentially placed, ensuring that their centroids and centers of gravity were aligned along the same plumb line.

(6) Setup of Displacement Sensors and Data Acquisition System: The displacement sensors were fixed to the reaction frame’s column using magnetic holders, ensuring that the probe of the displacement sensor made contact with the upper surface of the loading pad. The soil pressure cell, load sensor, and displacement sensor were connected to the data acquisition system, and a zero calibration was performed.

(7) The displacement sensor and connection data collection system were set up. The displacement sensor was fixed to the reaction frame column and a magnetic base was used. The probe of the displacement sensor was in contact with the surface of the loaded pad. The earth pressure box, load sensor, displacement sensor and JM3813 multi-function static strain collector were collected and the data was zeroed.

(8) Loading and Unloading: The loading and unloading method followed the procedure outlined in references [21,22,23], with 10 equal steps of loading until 85% of the soil pressure cell’s capacity was reached. Each loading step lasted for 1 min. Data was collected once the readings stabilized, and the next load step was applied. After the full load was reached, unloading began after 2 min. The unloading process followed the same procedure as the loading.

(9) Clearing the Sand: To ensure the same initial density of the sand for each test, after each experiment, the sand in the calibration barrel was removed. A brush was used to clean any residual sand on the side walls and film inside the barrel, and the weight of the sand was measured.

2. Interfaced Test Procedure

Unlike in the embedded test, in this procedure, an acrylic sheet was first placed at the bottom of the calibration container, and the soil pressure gauge was then inserted into the groove at the center of the acrylic sheet.

## 3. Basic Experiment

As mentioned in the introduction, sand, as a granular medium, exhibits distinct mechanical properties compared to homogeneous fluid media. Its stress history influences the output of the soil pressure gauge, leading to nonlinear and hysteretic features in the load-strain curve during loading, as well as residual strain (non-zero zero drift) after unloading. Moreover, with increasing load and sand compression modulus, as well as greater backfill thickness, both the nonlinear and hysteretic characteristics of the load-strain curve and the residual strain will change. To avoid the impact of these phenomena on the comparison and analysis of test results in subsequent factor analysis, it is necessary to first conduct a baseline calibration test. This will help determine the optimal number of loading cycles and the ideal backfill thickness in order to eliminate zero drift.

**Figure 5:**
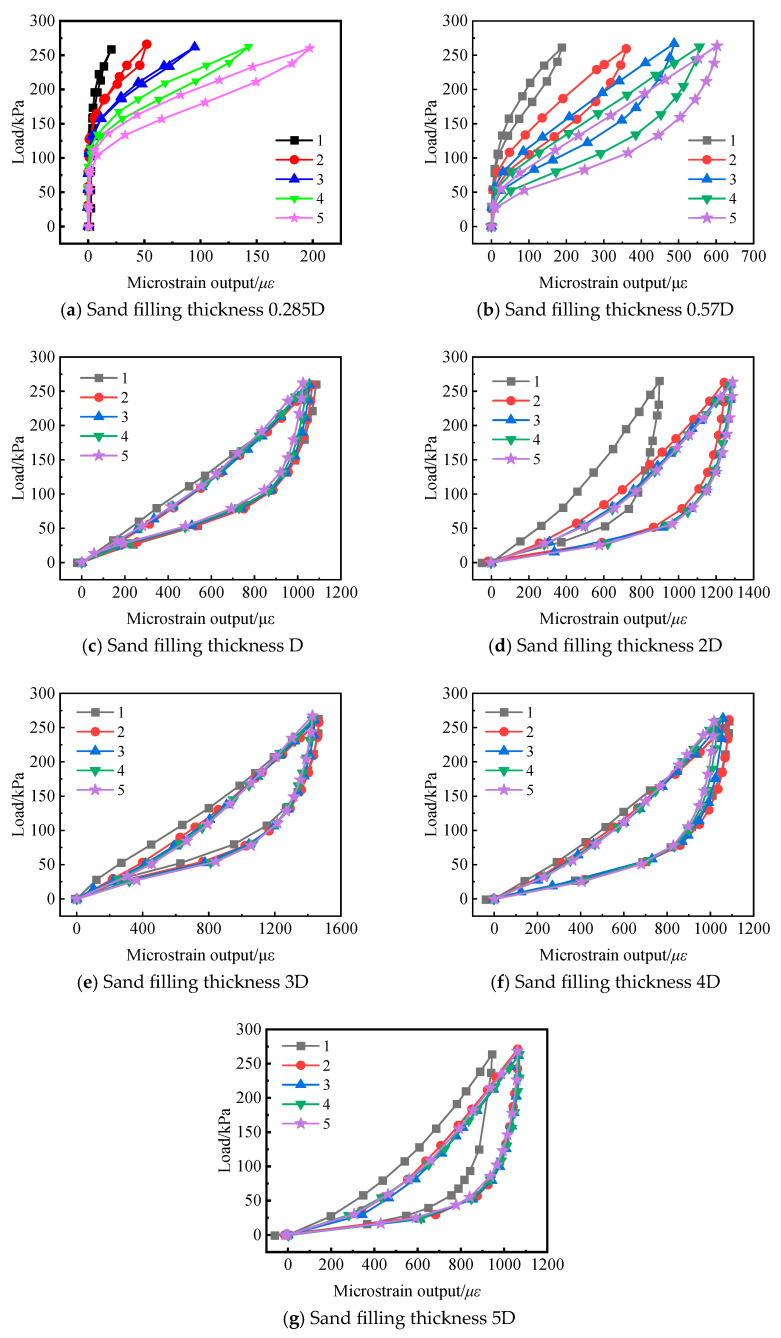
Calibration Curve of the Soil Pressure Cell.

The basic experiment employed an interface-based testing method, where the soil pressure cell was embedded in an acrylic board to ensure that the upper surface of the pressure cell was flush with the upper surface of the acrylic board. In the calibration test of different sand filling thicknesses, the thickness of the sand filling was set to 0.285D, 0.57D, D, 2D, 3D, 4D, and 5D for each group of tests. Based on preliminary pilot tests and a review of existing literature, the number of loading and unloading cycles was initially determined to be 5. Figure 5 presents the calibration curves of the soil pressure cell for the 7 sets of tests. As shown in the figure, the calibration tests with sand thicknesses of 0.285D and 0.57D exhibit significantly different loading and unloading curve characteristics compared to the other tests. This is similar to the experimental phenomena observed by Zeng Hui [24] and Trllope [5], where calibration tests with a fill thickness less than D are not recommended. The following analysis focuses on the remaining five sets of tests.

(1) Shape of the Calibration Curves: Except for the first cycle of loading and unloading, the calibration curves for the 2nd to 5th cycles almost coincide. This is mainly due to the progressive compaction of the sand during loading, which causes the sand’s compressive modulus to gradually increase and then stabilize at a certain value. The calibration curves exhibit hysteresis, with the loading and unloading curves not coinciding. This is primarily because the compressive modulus and the rebound modulus of the sand differ. In addition, the sensor diaphragm may not fully rebound, and friction between the calibration barrel’s inner wall and the diaphragm also has a minor impact on the measurements.

(2) As is shown in Table 2, after complete unloading, the output of the soil pressure cell is not zero, indicating that the zero drift value is not zero. The thicker the sand layer, the larger the zero drift value. As the number of loading and unloading cycles increases, the zero drift value decreases. When the number of loading and unloading cycles exceeds three, the zero drift value becomes zero.

In summary, for the factor analysis, the number of loading and unloading cycles for each set of tests was determined to be 5. The calibration curves from the 4th and 5th loading-unloading cycles were selected as the objects of analysis to ensure the validity of comparisons across the test groups.

(3) Nonlinearity of the calibration curve. When the sand filling thickness is 0.285D and 0.57D, both the loading curve and the unloading curve are nonlinear. Especially when the sand filling thickness is 0.285D the image of the calibration curve load is approximately y = ln (kx + b) (k, b > 0). As the number of loading times increases, the k value continues to decrease, and the nonlinearity of the curve gradually increases. When the sand filling thickness is 1D to 5D, the linearity of the loading curve is relatively strong, while the unloading curve is still nonlinear.

## 4. Analysis of Influencing Factors

As mentioned earlier, the following discussion will focus on the influence of various factors on the calibration results under different experimental conditions: sand thickness under both interface-based and embedded tests, sand particle size under interface-based tests, distance between soil pressure cells under embedded test conditions, and the embedding depth of the soil pressure cell under interface-based tests.

### 4.1. Effect of Sand Filling Thickness

#### 4.1.1. Upper Sand Filling Thickness of Interface Earth Pressure Cell

**Figure 6:**
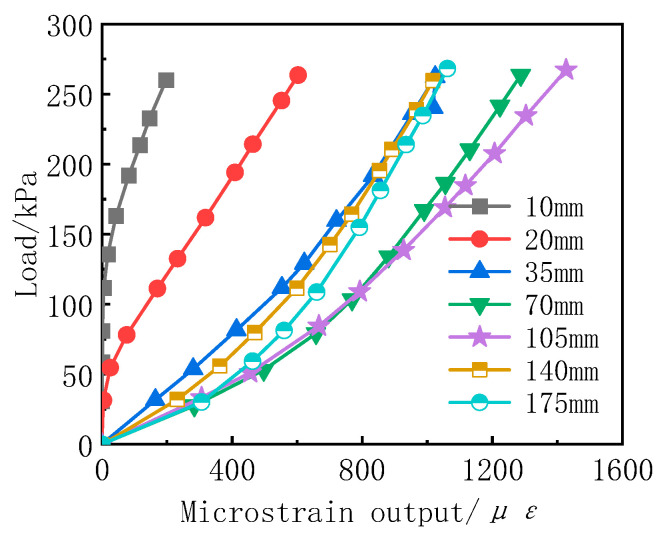
Calibration curves of the earth pressure cell with different sand filling thickness.

Figure 6 shows the calibration curves of the interface-type soil pressure cell under different sand fill thicknesses. As the sand fill thickness increases from 10 mm to 175 mm, the microstrain of the soil pressure cell first increases and then decreases, which is consistent with the experimental trends observed by Dave et al. [7]. When the sand fill thickness is less than 105 mm (3D), the microstrain of the soil pressure cell increases with the fill thickness, which is in good agreement with the experimental results of Gade et al. [9,10] and Cai Zhengyin et al. [11]. This is because the elastic modulus of the soil pressure cell differs from that of the acrylic material by nearly an order of magnitude. As the sand fill thickness increases, the stress concentration on the surface of the soil pressure cell becomes more pronounced, leading to an increase in the microstrain. When the sand fill thickness exceeds 105 mm (3D), the microstrain of the soil pressure cell slightly decreases with any further increase in fill thickness, which is consistent with the experimental trends reported by Li Chunlin et al. [8]. This is due to both the increase in side resistance between the sand and the barrel wall as the fill thickness increases and the soil arching effect, which transfers the load to the area surrounding the soil pressure cell, resulting in a reduction in microstrain.

As mentioned above, the differences in the mechanical properties between the soil pressure cell and the surrounding medium led to a continuous redistribution of stress in what would theoretically be a uniform stress field. The theoretical uniform stress can be obtained using a manometer. In calibration experiments, scholars often define the difference between the stress measured by the soil pressure cell and the theoretical uniform stress as the matching error [24], as shown in Equation (1).

(1) $$\beta =\frac{P_{t}-P_{0}}{P_{0}}=\frac{K_{o}\varepsilon -K_{s}\varepsilon }{K_{s}\varepsilon }=\frac{K_{o}}{K_{s}}-1$$

wherein β is the matching error, expressed as a percentage; *P*_t_ is the stress value measured by the soil pressure cell after the soil medium is disturbed, expressed in kPa; *P*_0_ is the true stress value before the soil medium is disturbed, expressed in kPa; *K*_0_ is the oil scale coefficient provided by the manufacturer, expressed in kPa/με; *K*_s_ is the sand scale coefficient, which is obtained by fitting a linear function y = *K*_s_x to the loading curve, expressed in kPa/με; *ε* is the microstrain output by the soil pressure cell, expressed in με.

**Figure 7:**
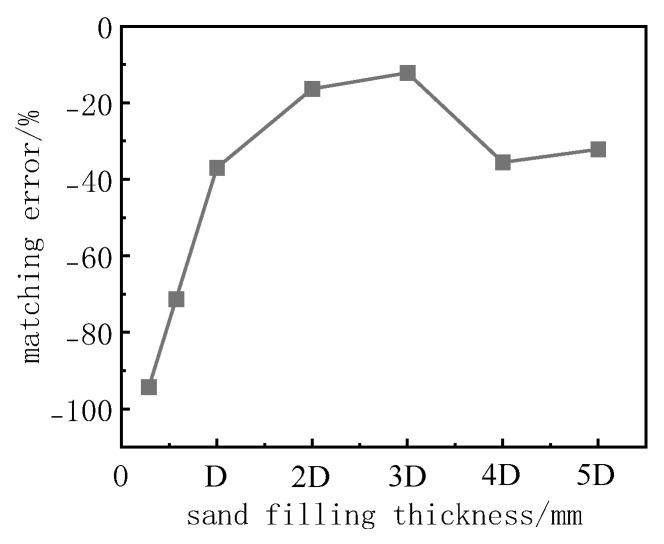
Relationship between matching error and sand filling thickness.

Figure 7 presents the matching error for each test group under different sand fill thicknesses, calculated using Equation (1). As shown in the figure, the matching errors for all test groups are negative, indicating that the measured values are smaller than the true values. Overall, as the sand fill thickness increases, the matching error first increases and then decreases. The minimum matching error corresponds to a sand fill thickness of 3D. This suggests that, for interface-type soil pressure cell sand calibration tests conducted with this setup, a sand fill thickness of 3D results in measurements that are closer to the theoretical values.

#### 4.1.2. Upper and Lower Sand Filling Thickness of Buried Earth Pressure Box

In order to study the range of stress fields affecting the upper and lower parts of the earth pressure box and the development of the soil arching effect, and to obtain the sand filling thickness with the smallest calibration test error, calibration tests of different upper and lower sand filling thicknesses of the buried earth pressure box were carried out. The sand filling thicknesses in the calibration bucket were set to 20 mm, 40 mm, 70 mm, 140 mm, 210 mm, and 280 mm. To ensure that the upper and lower sand filling thicknesses of the earth pressure box are equal, the upper and lower sand filling thicknesses of the earth pressure box were set to 10 mm, 20 mm, 35 mm, 70 mm, 105 mm, and 140 mm.

**Figure 8:**
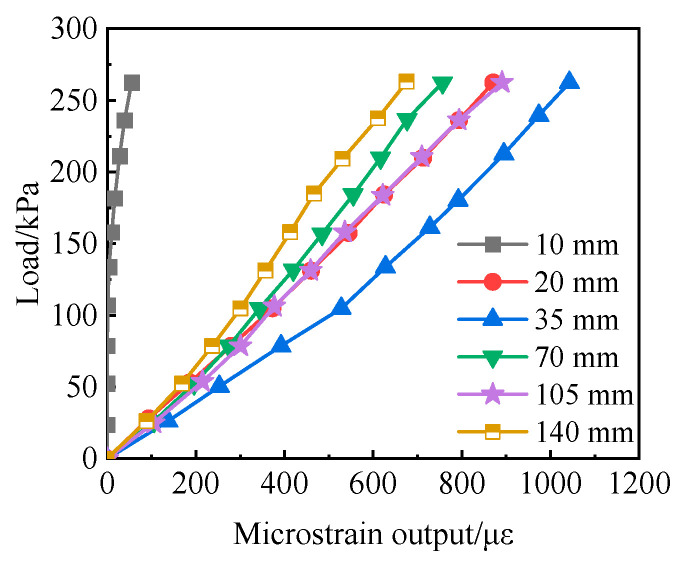
Calibration curves of the earth pressure cell with different sand filling thickness.

Figure 8 shows the calibration curve of the buried earth pressure box with different upper and lower sand filling thicknesses. It can be seen from the figure that, with the increase in sand filling thickness, the microstrain output by the earth pressure box first increases and then decreases. When the upper and lower sand filling thickness is 10 mm, the microstrain output by the earth pressure box is very small, and the nonlinearity of the calibration curve is strong.

**Figure 9:**
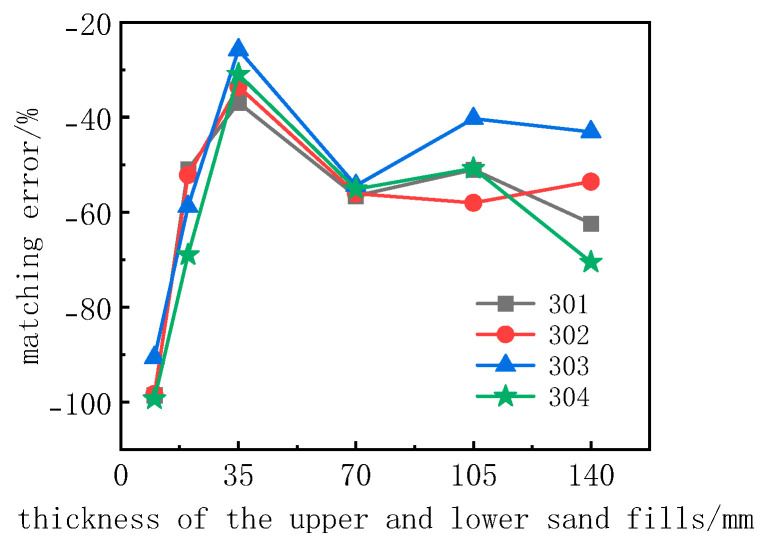
Curves of the matching error of the thickness of the upper and lower sand fills.

Figure 9 gives the test errors of the tests of the buried soil pressure cell. Given the curve of the thickness of the sand filling, it can be seen that the thickness of the sand filling from 10 mm to 140 mm (4D) is increased. When the thickness of the sand filling at the bottom of the soil pressure cell is 10mm, the matching error is the smallest, about −100%; when the thickness of the sand filling box is 140 mm (4D), the matching error is more than −60%; when the thickness is 35 mm (1D), the matching error of the soil pressure cell is about −30%, and the absolute value is the smallest. Our analysis found that, when the buried soil pressure cell is subjected to a calibration test, the thickness of the sand filling of the soil pressure cell can be set to 1D, which can effectively reduce the test error and improve the measurement accuracy of the soil pressure cell.

### 4.2. Effect of Sand Particle Size

**Figure 10:**
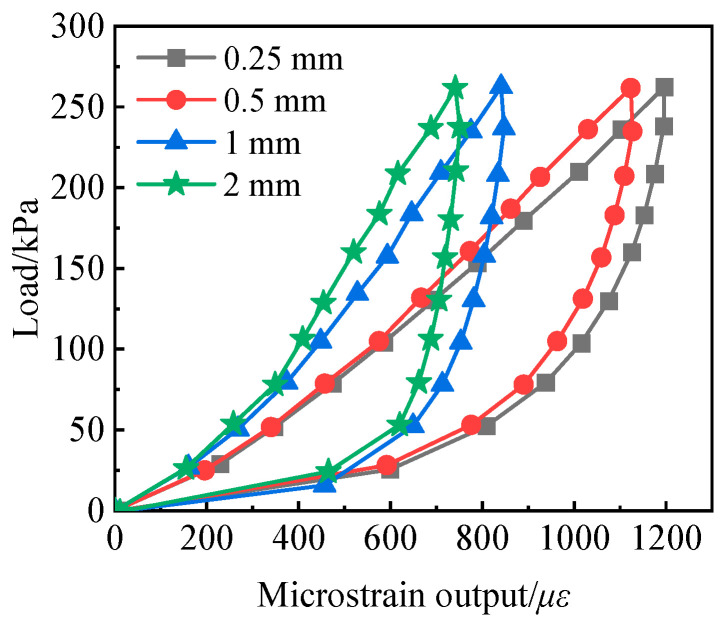
Calibration curves of the earth pressure cell with different sand particle size.

Based on the analysis of the effect of sand fill thickness, the influence of sand particle size on the calibration results of the soil pressure cell was further investigated through interface-type experiments. As shown in Table 1, with all other parameters kept constant and only the sand particle size varied, Figure 10 presents the calibration curves of the soil pressure cell for different sand particle sizes. It can be observed from the figure that the smaller the sand particle size, the greater the microstrain output of the soil pressure cell under the same load. This is because with larger sand particles, the soil arching effect becomes more significant after loosening and compaction of the sand. Furthermore, the smaller the sand particle size, the more uniformly the sand distributes its force across the sensing diaphragm of the soil pressure cell, leading to a higher microstrain output from the soil pressure cell.

Zeng Hui et al. [24] (2005) analyzed the interaction between the earth pressure cell and its surrounding soil medium and proposed a theoretical formula for the matching error of the single-membrane earth pressure cell for measuring the surface stress of the structure based on the double collapse model, as shown in Formula (2).

(2) $$\beta =\frac{P_{t}-P_{0}}{P_{0}}=\frac{\frac{d}{D}\cdot \frac{E_{g}}{E_{c}}-1}{0.191\left(1-{\mu_{s}}^{2}\right)\cdot \frac{d}{H}\cdot \frac{E_{g}}{E_{s}}+1}$$

wherein β is the matching error, expressed as a percentage; *P*_t_ is the stress measured by the soil pressure cell in kPa; *P*_0_ is the theoretical uniformly distributed stress in kPa; D is the diameter of the soil pressure cell in mm; d is the diameter of the sensing diaphragm in mm; *H* is the thickness of the soil pressure cell in mm; *E*_g_ is the equivalent deformation modulus of the soil pressure cell in kPa; *E*_s_ is the compression modulus of the soil in kPa; *E*_c_ is the elastic modulus of the structure in kPa; *μ*_s_ is the Poisson’s ratio of the soil.

**Figure 11:**
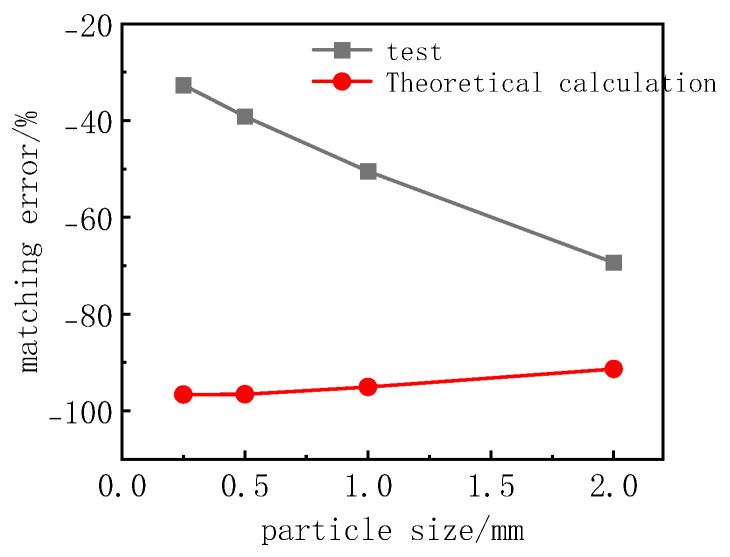
Relationship between matching error and sand particle size.

Figure 11 gives the theoretical value of the matching error based on the compressed modulus of each particle size of sand. The test values are large, with the particle size of the sand and the law of the experimental value and the theoretical value being opposite to the law of the particle size of the sand. It is not difficult to infer that, for the changes in the size of the messenger particle, the mixing error was caused by different loads on the loading point of the soil pressure cell accounting for the leading role.

Based on Zeng Hui’s theoretical formula, this paper further derives the matching error theoretical formula of embedded and interface earth pressure cells by combining the difference between the matching error test value and Zeng Hui’s theoretical calculation value.

(3) $$\beta_{1}=\frac{\frac{d}{D}\cdot \frac{E_{g}}{E_{c}}-1}{f\left(T/D\right)f\left(d/d_{smax}\right)\cdot \frac{\left(1-{\mu_{s}}^{2}\right)}{4}\cdot \frac{d}{H}\cdot \frac{E_{g}}{E_{s}}+1}$$

(4) $$\beta_{2}=\frac{\frac{d}{D}\cdot \frac{E_{g}}{E_{s}}-1}{f\left(T/D\right)f\left(d/d_{smax}\right)\cdot \frac{\left(1-{\mu_{s}}^{2}\right)}{4}\cdot \frac{2d}{H}\cdot \frac{E_{g}}{E_{s}}+1}$$

In Formulas (3) and (4), *T* is the sand filling thickness (burial depth) in mm; d_smax_ is the maximum particle size of sand in mm.

**Figure 12:**
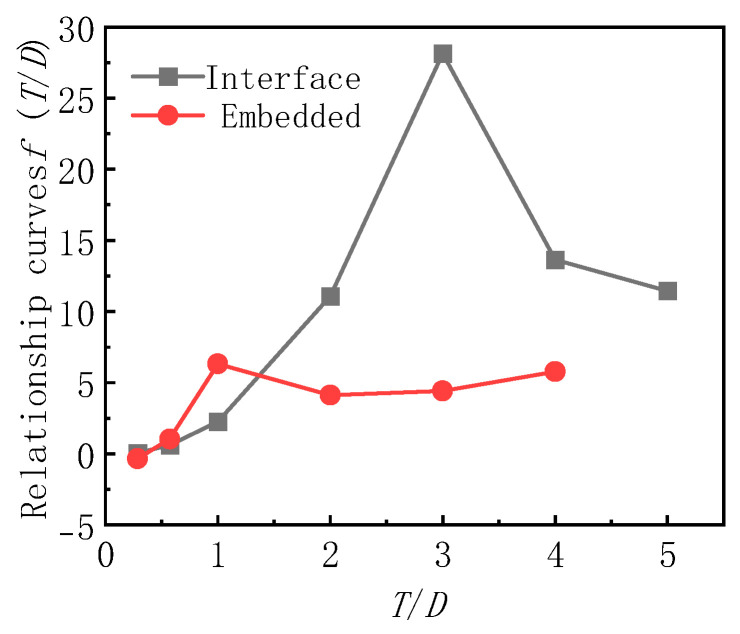
Relationship curves of f(T/D) and T/D.

**Figure 13:**
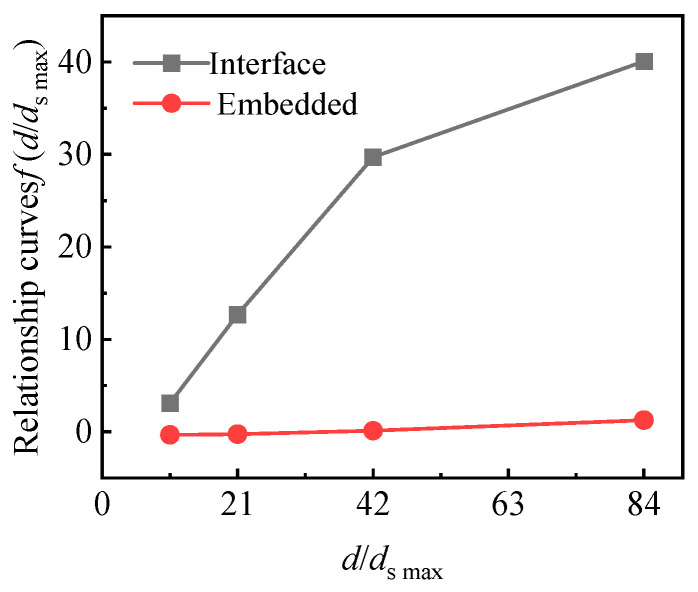
Relationship curves of $f(d/d_{smax})$ and $d/d_{smax}$.

The relationship curves of f(T/D) and T/D are shown in Figure 12, and the relationship curve of $f(d/d_{smax})$ and $d/d_{smax}$ is shown in Figure 13.

Table 3 and Table 4 are the fitting functions corresponding to the curves in Figure 12 and Figure 13, respectively. The analysis shows that, when the thickness of the upper sand filling of the interface earth pressure box is greater than 3D and the thickness of the upper and lower sand filling of the buried earth pressure box is greater than 1D, f(T/D) is a constant.

### 4.3. Effect of Depth Inserted in the Structure

**Figure 14:**
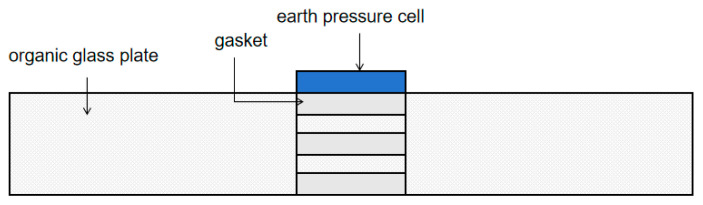
Schematic diagram of changing the depth of the earth pressure cell inserted in the plexiglass plate.

In large-scale model tests, soil pressure cells are often embedded within the structure to measure soil pressure. However, the effect of the embedding depth of the soil pressure cell on the measured soil pressure remains unclear. To address this, the embedding depths of the soil pressure cells were set at −H, −0.5H, 0, +0.5H, and +H, as shown in Table 1, in order to investigate the impact of embedding depth on the calibration results through interface-based tests. In Table 1, the embedding depths of “−”, “+”, and “0” correspond to the situations where the sensing surface of the pressure cell is below, above, or level with the upper surface of the acrylic plate, respectively. Here, H represents the thickness of the acrylic plate. Figure 14 illustrates the embedding of the soil pressure cell in the acrylic plate.

**Figure 15:**
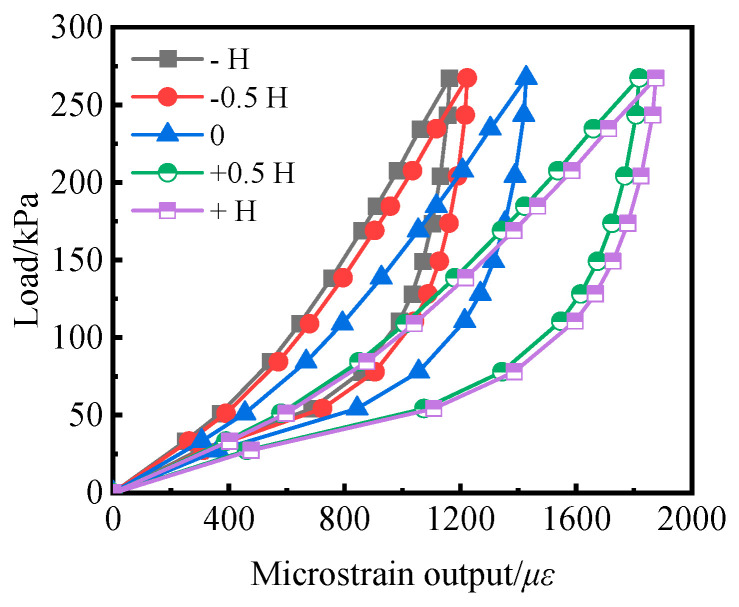
Calibration curves of the earth pressure cell with different inserted depths.

The embedded depth of the soil pressure cell is set to −H, −0.5H, 0, +0.5H, and +H, and the embedded depth to “−”, “+”, and “0”, respectively. The sensor surface is higher than the inductive surface on the surface of the organic glass plate. The calibration curve of each soil pressure cell is shown in Figure 15. Under the conditions of the same level, the embedded depth of the soil pressure cell increases from −H to +H (the soil pressure cell rises from the organic glass plate groove to the surface of the protruding organic glass plate). The strain gradually increased.

**Figure 16:**
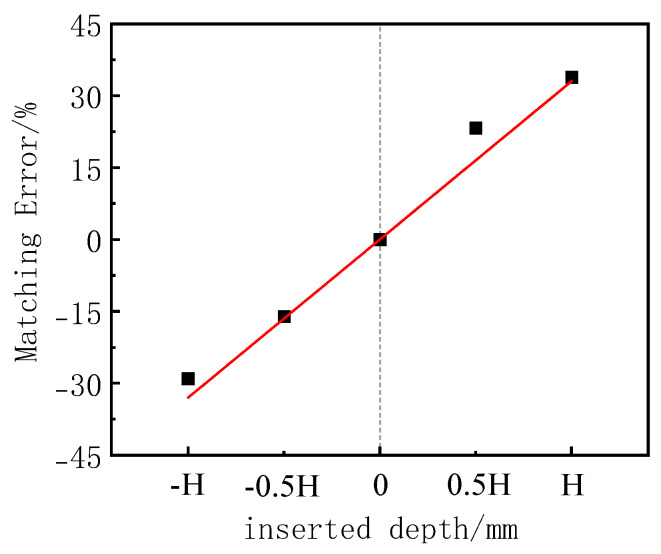
Fitting relationship between matching error and inserted depth.

We used Formula (1) to calculate the matching error of each group of tests, and the test matching error of the soil pressure cell and the mitigation curve of the embedded depth are shown in Figure 16. When the induction surface of the soil pressure cell is lower than the surface of the organic glass plate, the matching error is negative, indicating that the measurement value is small; when the induction surface of the soil pressure cell is higher than the surface of the organic glass plate, the matching error is positive, indicating that the measurement value is large; when the surface of the glass plate is flat, the matching error is close to 0. Therefore, when the soil pressure cell is used to measure the surface stress of the structure, the induction surface and the structure surface of the soil pressure cell should be ensured. The results of this test reached an agreement with the numerical simulation results of [9] and Rusinek and others [19], as the calibration test results of the calibration medium matched the calibration medium.

The function of the fitting curve In Figure 16 Is shown In Formula (5). If the sensing surface of the earth pressure box is not completely flush with the structure surface, the corresponding matching error can be calculated by this formula and the earth pressure measurement value can be corrected.

(5) $$\beta =33\frac{z_{v}}{H}$$

wherein β and *H* are the same as before; *z*_v_ is the depth of the earth pressure box embedded in the structure, expressed in mm.

### 4.4. Horizontal Spacing of the Soil Pressure Cell

**Figure 17:**
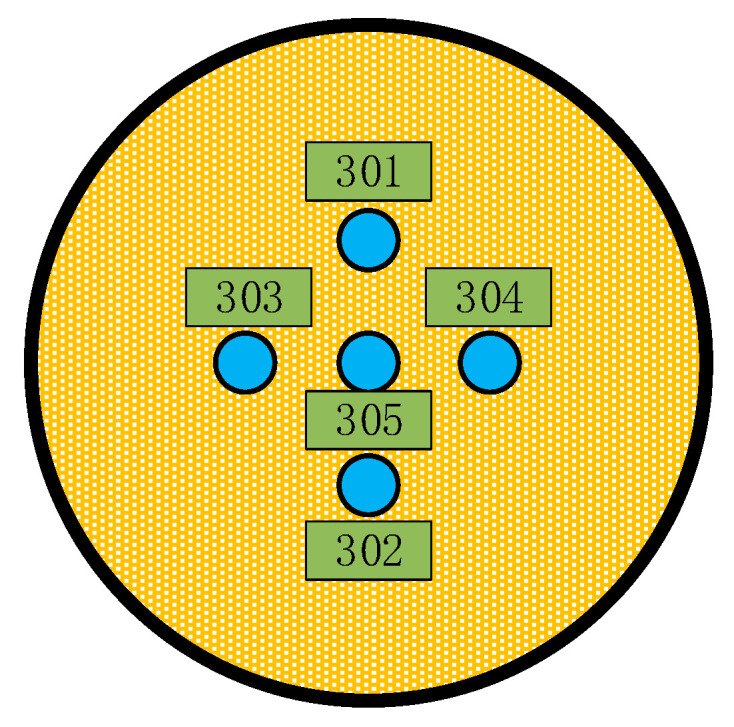
Horizontal distribution of earth pressure cells in a free field of sand.

In the previous experiments, only one soil pressure cell was calibrated per test, which resulted in low testing efficiency. To improve this efficiency, the following analysis aims to investigate the effect of the horizontal spacing between soil pressure cells on the calibration results when multiple soil pressure cells are calibrated simultaneously in a single test. Figure 17 illustrates the layout of the soil pressure cells in the embedded test, where the soil pressure cells are numbered 301, 302, 303, 304, and 305. During the test, the horizontal spacing between the cells numbered 301, 302, 303, 304 and the cell numbered 305 was varied simultaneously. The specific parameters are provided in Table 1.

**Figure 18:**
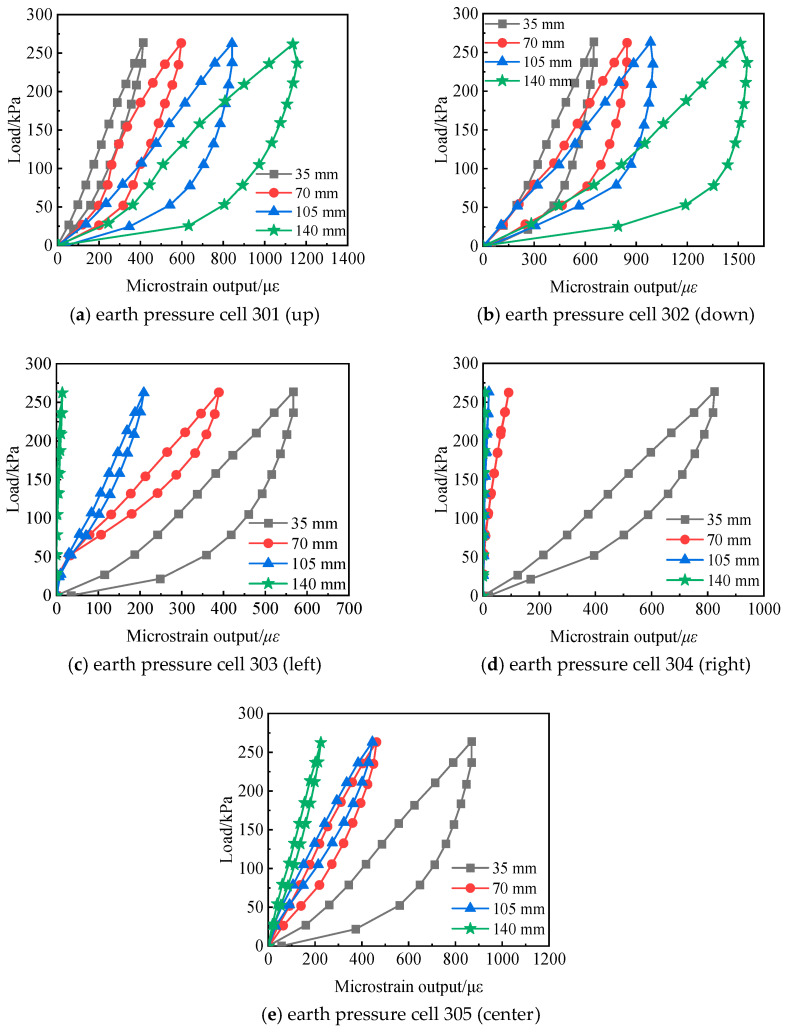
Calibration curves of earth pressure boxes at different locations.

Figure 18 gives the calibration curve of the soil pressure cell when in different positions. It can be seen that, under the condition of the same load, the soil pressure cells in the upper, lower, and right positions spread around. Largely, the micro-changes in the outputs of the soil pressure cells in the upper and lower positions gradually increase, and the stagnation of the calibration curve gradually increases. When the left and right position soil pressure cells are spaced 140 mm (4D) from the center point of the calibration barrel, and the distance between the shell of the soil pressure cell and the inner wall of the calibration barrel is 42.5 mm (1.2D), as the load is increased, the output of the soil pressure cell is on the micro scale. The strain is almost unchanged, and the calibration curve has no stagnation.

In summary, it is currently not possible to calibrate multiple earth pressure boxes on the same horizontal plane at the same time. It is recommended to place a single earth pressure box at the center of the calibration barrel for a calibration test, to or use the test matching error to correct the calibration results of the earth pressure boxes distributed around the barrel, and thereby indirectly obtain the calibration results of the earth pressure box at the center.

## 5. Discussion

Soil pressure measurement methods and results primarily depend on the experimental conditions. When the soil pressure cell is embedded in the soil or integrated into the surface of a structure, it can measure the pressure in the soil. Whether the measurement is static or dynamic depends entirely on the engineering object. Currently, soil pressure cell calibration tests mainly aim to correct the measurements under relatively static conditions. For example, this includes measurements of soil pressure behind retaining structures in excavation projects, soil pressure at the top of piles in pile foundations, and the surrounding pressure around tunnels. The measurement issues raised by experts regarding soil pressure during shield tunneling, considering the vibration from the cutter head cutting through rock and soil, which can affect the measurement accuracy, and the irregularity of the excavation face, which further reduces accuracy, will be addressed in future soil pressure calibration tests. In practical engineering or indoor model tests, factors such as the size of soil particles, the thickness of soil layers, and the installation methods of the soil pressure cells (such as whether they are embedded or surface-mounted) can cause significant discrepancies between the measured and actual soil pressure values. To correct this, before using the soil pressure cells, a calibration test can be performed to determine the matching error coefficient, which can then be multiplied by the measured values to obtain the true pressure. For example, this study suggests that for interface-type and embedded soil pressure cell calibration tests, the optimal fill thicknesses should be 1D and 3D, respectively, with the number of loading-unloading cycles being greater than 4. Each test group should calibrate only one soil pressure cell. These recommendations can serve as a reference for calibrating soil pressure cells. Moreover, this study also proposes using the matching error correction formula to adjust the soil pressure measurements on both sides in model tests. It is recommended that the sensing surface of the soil pressure cell should be flush with the surface of the structure. When it is not possible to align the sensing surface of the soil pressure cell with the surface of the acrylic plate in scale model tests, the correction formula presented in this paper can be used to adjust the soil pressure measurement results.

## 6. Summary and Conclusions

In order to improve the measurement accuracy of the single-membrane resistive soil pressure gauge on structural surfaces during model tests, this study primarily conducted interface-type experiments, supplemented by embedded-type experiments. The effects of factors such as sand thickness, grain size, the horizontal distance of the soil pressure gauge, and embedding depth on the calibration results were systematically investigated. Additionally, the relevant calculation theories for existing matching errors were supplemented and revised. The preliminary conclusions applicable to this experimental setup are as follows:

For the interface-type and embedded-type calibration tests of soil pressure gauges, it is recommended that the optimal fill thickness be 1D and 3D, respectively. The number of load-unload cycles should exceed four, and it is advisable to calibrate only one soil pressure gauge per test group.

For the interface-type calibration test, the smaller the grain size of the sand, the greater the microstrain output from the soil pressure gauge under the same load level. In the matching error calculation analysis, in addition to considering the compressive modulus and Poisson’s ratio of the sand, the elastic modulus of the structure, the diameter of the soil pressure gauge, the diameter of the sensing diaphragm, and the thickness of the soil pressure gauge, the effects of sand thickness and grain size should also be taken into account. The formula proposed in this paper can be used to correct the soil pressure measurement results in scale model tests.

In the interface-type calibration test, when the sensing surface of the soil pressure gauge is below the upper surface of the acrylic plate, the matching error is negative, indicating that the measured value is lower than the true value. When the sensing surface of the soil pressure gauge is above the upper surface of the acrylic plate, the matching error is positive, indicating that the measured value is higher than the true value. When the sensing surface of the soil pressure gauge is flush with the upper surface of the acrylic plate, the matching error is close to zero. It is recommended that, in scale model tests, the sensing surface of the soil pressure gauge be flush with the structural surface. If it is not possible to align the sensing surface of the soil pressure gauge with the upper surface of the acrylic plate during the test, the formula proposed in this paper can be used to correct the soil pressure measurement results.

## Figures and Tables

**Table 1:** Specification of calibration tests.

Test Item	Calibration Medium		Thickness of the Sand Layer		Interface/Embedded	The Depth Inserted in the Structure/mm	The Position of the Soil Pressure Cell/mm
			Thickness of the Topsoil/mm	Thickness of the Backfill/mm			
1	Standard sand		10 (0.285D)	10 (0.285D)	Embedded	/	Each test group is calibrated with only one soil pressure gauge
			20 (0.57D)	20 (0.57D)			
			35 (1D)	35 (1D)			
			70 (2D)	70 (2D)			
			105 (3D)	105 (3D)			
			140 (4D)	140 (4D)			
			175 (5D)	175 (5D)			
			10 (0.285D)	/	Interface	0	
			20 (0.57D)	/			
			35 (1D)	/			
			70 (2D)	/			
			105 (3D)	/			
			140 (4D)	/			
			175 (5D)	/			
2	Different particle size sand	1 mm~2 mm	35 (1D)		Embedded	/	
		0.5 mm~1 mm					
		0.25 mm~0.5 mm					
		<0.25 mm					
		1 mm~2 mm	105 (3D)		Interface	0	
		0.5 mm~1 mm					
		0.25 mm~0.5 mm					
		1 mm~2 mm					
3	Standard sand		35 (1D)	35 (1D)	Embedded	0	0
							70 (2D)
							140 (4D)
4	Standard sand		105 (3D)	/	Interface	−6	Only one soil pressure gauge

Note: (1) “D” in the table is the diameter of the earth pressure cell; (2) in the fourth test, “−” means that the sensing surface of the earth pressure cell was lower than the upper surface of the plexiglass plate; (3) ‘/’ denotes that the parameter was not considered.

**Table 2:** Zero drift values of calibration curves under different sand thicknesses and stepwise loading conditions.

Loading and Unloading Cycles	Sand Filling Thickness/mm						
	10	20	35	70	105	140	175
1	0	0	9	22	40	51	61
2	0	0	1	1	7	17	14
3	0	0	0	0	3	3	3
4	0	0	0	0	0	0	0
5	0	0	0	0	0	0	0

**Table 3:** The fitting function related to the thickness of the sand fill.

Buried Method	Sand Filling Thickness	Fitting Function	Goodness of Fit R^2^
Interface	*T* ≤ 3D	f(T/D)=10.273T/D−5.666	0.9349
	*T* > 3D	f(T/D)=12.542	/
Embedded	*T* ≤ 1D	f(T/D)=9.559C/D− 3.577	0.9565
	*T* > 1D	f(T/D)=4.772	/

**Table 4:** The fitting function related to the maximum particle size of the sand.

Buried Method	Sand Filling Thickness	Fitting Function	Goodness of Fit R^2^
Interface	*T* = 3D	$f(d/d_{smax})=0.488d/d_{smax}+\;2.18$	0.9075
Embedded	*T* = 1D	$f(d/d_{smax})=0.022d/d_{smax}-0.692$	0.9758

## Data Availability

No new data were created. Data sharing is not applicable in this paper.
